# Osseointegration in the Absence of Primary Stability: An Experimental Preclinical Mandibular Minipig Overpreparation In Vivo Model

**DOI:** 10.1111/clr.70006

**Published:** 2025-07-23

**Authors:** Thomas Gill, Hansley Ooi, Emre Tezulas, Aviva Petrie, Simon Rawlinson, Mario Roccuzzo, Shakeel Shahdad

**Affiliations:** ^1^ Institute of Dentistry, Barts and the London School of Medicine and Dentistry Queen Mary University of London London UK; ^2^ Department of Restorative Dentistry, the Royal London Dental Hospital Barts Health NHS Trust London UK; ^3^ Private Practice Penang Malaysia; ^4^ Department of Restorative Dentistry, Faculty of Dental Surgery University of Malta Msida Malta; ^5^ Faculty of Medical Sciences, Eastman Dental Institute University College London London UK; ^6^ Private Practice Torino Italy; ^7^ Department of Maxillo‐Facial Surgery University of Torino Torino Italy; ^8^ Department of Periodontics and Oral Medicine University of Michigan Ann Arbor Michigan USA

**Keywords:** BIC, bone‐implant‐contact, crestal bone formation, implant, implant surface, in vivo, osseointegration, primary stability

## Abstract

**Objectives:**

The effect of osteotomy overpreparation, and thus lack of primary stability, on implant osseointegration and crestal bone volume maintenance was investigated by comparing placement of dental implants with either a standard osteotomy preparation (NP) or an overprepared osteotomy (OP) where the final osteotomy drill was larger in diameter than the implant placed.

**Methods:**

Bone‐level implants (Ø3.3 mm diameter) were placed in the mandible of minipigs with two preparation techniques: an NP (Group 1) and an OP to a final osteotomy of 3.5 mm in diameter (Group 2) and submerged for 2 and 8 weeks. An Implant Stability Quotient (ISQ) was measured for each implant at placement. Implant survival, defined histologically as the absence of fibrous encapsulation and the presence of direct bone‐to‐implant contact, osseointegration and crestal bone formation were analysed histologically and histomorphometrically to compare the preparation techniques.

**Results:**

A 100% survival for both preparation types was observed. The mean ISQ at insertion for Groups 1 and 2 was 69.35 a.u. (95% CI: 68.02–70.68) and 11.95 a.u. (95% CI: 10.53–13.37) respectively (*p* < 0.001). At 2 and 8 weeks, there was no difference between the two groups for total bone‐to‐implant contact (tBIC) (*p* > 0.05). Group 2 demonstrated significantly higher mean first bone‐to‐implant contact (fBIC), coronal bone‐to‐implant contact (cBIC) and bone‐area‐to‐total‐area (BATA) at 2 and 8 weeks compared to Group 1 (*p* < 0.05).

**Conclusion:**

Implants inserted into an overprepared osteotomy with no primary stability successfully osseointegrated. At 2 and 8 weeks, OP resulted in significantly more coronal bone apposition and maintenance of coronal bone volume as measured by fBIC, cBIC and BATA.

## 1 Introduction

Achieving primary stability at implant insertion is considered a prerequisite for osseointegration (Lioubavina‐Hack et al. 2006). The absence of primary stability that permits macromotion has been reported to result in fibrous encapsulation and, therefore, failure rather than osseointegration (Lioubavina‐Hack et al. 2006; Szmukler‐Moncler et al. 1998). A tolerable threshold of micromotion to permit osseointegration is proposed to range from 50 to 150 μm (Szmukler‐Moncler et al. 1998). In immediately loaded cases, high primary stability is an absolute requirement and has been associated with higher implant survival (Javed et al. 2013; Darriba et al. 2023).

Primary stability is the mechanical anchorage achieved through the physical congruence between the surgically created bone bed (osteotomy) and the implant, resulting in the absence of mobility when the implant is inserted (Albrektsson et al. 1986, 1981). Primary stability is multi‐factorial and a composite measure associated with bone quality, bone quantity, implant macro‐design (length, diameter, surface characteristics) and the surgical technique (Meredith 1998). On the contrary, secondary stability (true osseointegration) develops from regeneration and remodeling of the bone around the implant after healing (Sennerby and Roos 1998).

Surfaces that minimise the critical healing phase, where implants may be more susceptible to increased micromotion due to osteoclastic activity, by enhancing the rate of bone apposition have been developed (Schwarz, Ferrari, et al. 2007; Schwarz, Herten, et al. 2007; Lang et al. 2011; Smeets et al. 2016). Furthermore, specific surgical protocols have been developed, such as undersized osteotomies, with various implant geometries to increase primary stability at insertion (Wilson Jr et al. 2016; Sugiura et al. 2019; El Chaar et al. 2021). This has led to the consensus that to compensate for the biological reduction in implant stability, achieving high primary stability at insertion will provide mechanical anchorage to continue to resist movement despite osteoclastic remodelling, and permit robust secondary stability through osseointegration (Raghavendra et al. 2005).

Surrogate measures of primary stability have been studied and correlated with clinical outcomes to aid clinical protocol development and provide thresholds for treatment decisions (Atsumi et al. 2007). Two of the most reported non‐invasive and reliable quantitative measures of primary stability are resonance frequency analysis (RFA) and insertion torque (IT). IT provides a single measurement during implant insertion of the frictional resistance to movement along its axis through a rotational movement (Norton 2017). RFA measures the resonance frequency of the stiffness of the implant–bone complex by evaluating the implant stability and bone density using vibration analysis and can be quantified at multiple timepoints (Akkocaoglu et al. 2005; Turkyilmaz et al. 2009). Clinically imperceptible changes in stability can be detected with this method (Meredith 1998). However, there is no clear definition of the absence of primary stability as measured by RFA, posing the question of whether there is a threshold that should be met to permit implant placement, or conversely, below which an implant should not be placed?

Interestingly, clinical studies reporting low primary stability measurements in non‐immediately loaded implants have demonstrated high levels of implant survival comparable to implants placed with high‐medium stability measurements (Bischof et al. 2004; Schnitman and Hwang 2011; Ho et al. 2013; Berardini et al. 2016; Norton 2017; Lee et al. 2019). Although previous pre‐clinical studies have indicated the need for primary stability, recent clinical studies juxtapose this perception, with the exact relationship between primary stability and successful osseointegration remaining unclear in non‐immediately loaded clinical scenarios.

Therefore, further investigation in a preclinical model with an oversized osteotomy that permits movement of the implant is needed to allow a direct comparison of osseointegration to a conventionally placed implant that achieves primary stability. The aims of this non‐randomised controlled experimental in vivo study were to (i) assess if implants placed with low primary stability as measured by ISQ osseointegrate and (ii) histologically compare the quality of osseointegration between implants placed with high and no primary stability.

## 2 Material and Methods

### 2.1 Study Design

This controlled preclinical study aimed to investigate the effect of an overprepared osteotomy resulting in a lack of any primary stability on implant survival and quality of osseointegration. A mandibular minipig model using a single endpoint at 2 and 8 weeks after implantation was chosen as the test system. The impact of preparation type on osseointegration was investigated by comparing a standard preparation following the manufacturer's protocol (Group 1/Normal Preparation/NP) with an overprepared preparation resulting in osteotomy diameter larger than the implant's maximum diameter (Group 2/Overpreparation/OP). A moderately rough super‐hydrophilic surface (modSLA) and its relatively hydrophobic version (SLA) were chosen to allow assessment of the effect of preparation for each surface individually. The primary objective of this study was to determine if implants placed with low primary stability as measured by ISQ osseointegrated; defined as the absence of fibrous encapsulation and the presence of direct bone‐to‐implant contact histologically. The secondary objective of this study was to histologically assess the osseointegration and bone volume maintenance for implants placed without primary stability compared to conventionally placed implants for both SLA and modSLA surfaces.

Two test implants were utilised:

1. SLA—BL ø3.3 mm NC, Roxolid SLA 8 mm (Institut Straumann AG, Switzerland),
2. modSLA—BL ø3.3 mm NC RoxolidSLActive 8 mm (Institut Straumann AG, Switzerland).

**FIGURE 1:**
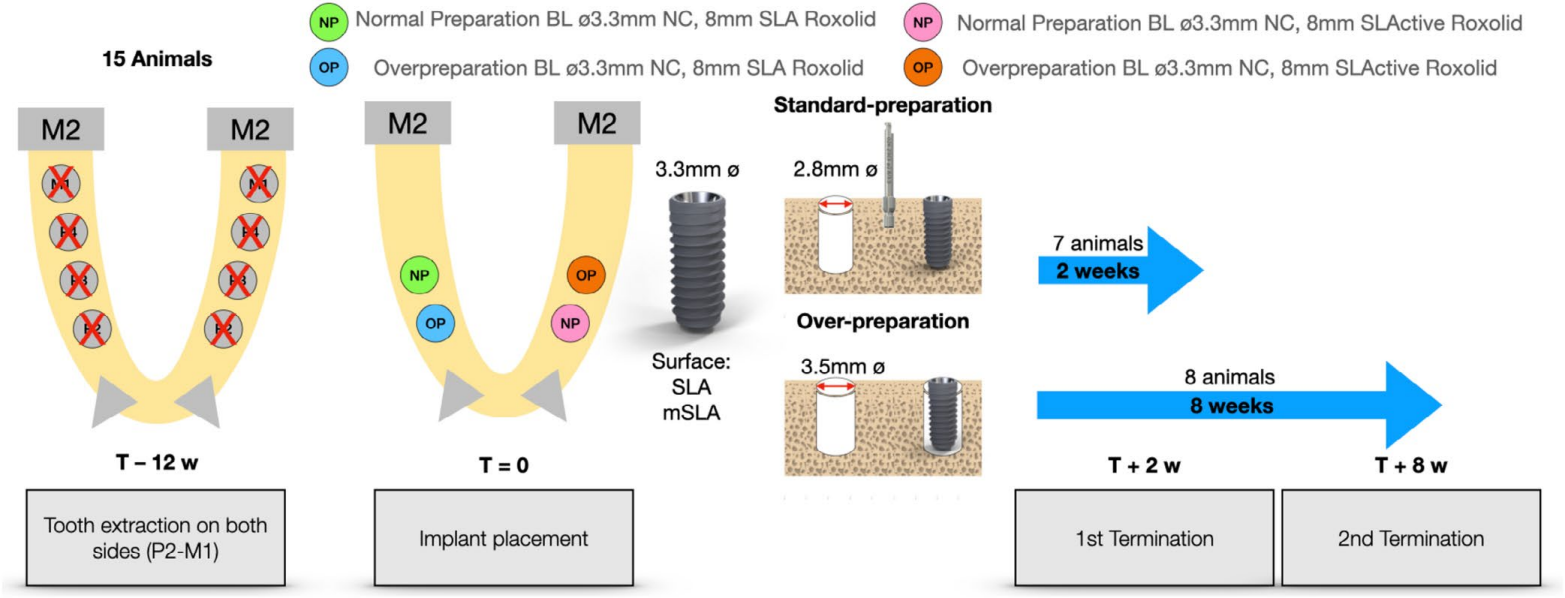
Overall study design. Overview of surgical timeline, implantation scheme and preparation types. Each hemi‐mandible received the same test implant (modSLA or SLA) with one preparation type (Group 1—Normal Preparation or Group 2—Overpreparation).

A total of 15 Göttingen Minipigs, 7 animals for the 2‐week timepoint and 8 animals for the 8‐week time point, were included in this study (Figure 1). Each hemi‐mandible received two adjacent implants with the same surface, such that one hemi‐mandible received SLA surface implants and the contralateral side modSLA surface implants. The preparation types were distributed between animals using a rotation scheme to ensure each preparation type was maximally represented at either anatomical position across the animals for each healing period.

To reduce the number of animals used for research, each animal received three additional implant groups, resulting in a total of 6 implants per animal that were part of a different study (Shahdad et al. 2022). This study was conducted at the Biomedical Department of Lund University (Lund, Sweden) and approved by the local Ethics Committee of the University (M‐192‐14), following the proper institutional and national guidelines for the care and use of the animals in the study. This study adhered to the ARRIVE 2.0 Guidelines and was designed by considering the 3R principle for animal research (Percie du Sert et al. 2020).

A power calculation determined that, to detect a mean difference of 15% in bone‐to‐implant contact and 0.75 mm in the first bone‐to‐implant contact—based on the standard deviations reported by Ou et al. (2016) and Imber et al. (2024) of 9.1 and 0.45 respectively, 7 animals with 2 of each group per animal would be required. This calculation assumed a paired *t‐*test comparison with 2 implants per animal in each group, a power of 80%, a significance level of 5% and included a 15% Wilcoxon adjustment (Lehmann 1998). Based on data from previous pre‐clinical studies using the mandibular minipig model, and balancing ethical considerations, anticipated attrition and the research team's prior experience, the final sample size was estimated to be 7 animals for the 2‐week timepoint and 8 for the 8‐week timepoint (Friedmann et al. 2014; Heuzeroth et al. 2021; El Chaar et al. 2021).

### 2.2 Animals

Fifteen female Göttingen Minipigs (Ellegaard) aged between 20 and 24 months at the time of surgery and an average body weight of 40 kg were included in the study. A mandibular minipig model in Göttingen Minipigs was selected for this study as it has been shown to most effectively reproduce osseointegration and alveolar bone remodelling around dental implants to that of humans (Musskopf et al. 2022). The animals were housed in standard boxes in groups of three. Animals were adapted to experimental conditions by starting animal housing 1 week before intervention. Animals were fed a standard soft food diet (Special Diet Services [SDS], Witham, UK #801586). Animals were fasted overnight before surgery to prevent vomiting.

### 2.3 Surgical Procedure

All surgical procedures were performed under general anaesthesia using a combination of dexmedetomidine (25–35 μg/kg i.m., Dexdomitor; Orion Pharma Animal Health) and tiletamine‐zolazepam (50–70 mg/kg i.m., Zoletil 100 Vet, Virbac) injected intramuscularly and maintained with intravenous infusion after induction with propofol (PropoVet multidose, Orion Pharma Animal Health) and fentanyl (Fentanyl B. Braun). Carprofen (4 mg/kg, s.i.d., i.m., Rimadyl vet., Orion Pharma Animal Health) was given as a preemptive dose and post‐surgically up to 4 days together with buprenorphine (0.03 mg/kg, i.m., Vetergesic vet, Orion Pharma Animal Health). To reduce the dosage of the systemic anaesthetic, bleeding during surgery, and to alleviate post‐surgical pain, local anaesthesia was provided intraoperatively by infiltrative injection of 1.8 mL of Xylocaine (Xylocaine, Dental adrenalin, 20 mg/mL and 12.5 μg/mL; Astra AB) per hemi‐mandible. Antibiotic prophylaxis was administered using benzylpenicillin prokain‐dihydrostreptomycin (25 mg/kg + 20 mg/kg, s.i.d, i.m., Streptocillin vet., Boehringer Ingelheim Vetmedica). Animals were intubated and breathing was withheld by a ventilator. Vital parameters were monitored continuously (pulse oximetry, rectal temperature, blood pressure, CO_2_).

Two surgical interventions were performed in each animal:

1. Extraction of mandibular premolars and molars (P2‐P4, M1).
2. Osteotomy creation and implant placement.

### 2.4 Tooth Extraction

Three contralateral mandibular premolars (P2–P4) and first mandibular molars (M1) were carefully extracted without raising a flap using a minimally invasive surgical approach.

### 2.5 Implant Osteotomy and Implant Placement

Implants were placed 20 weeks after extraction. Mandibular alveolar ridges were exposed by elevating a mucoperiosteal flap after a midcrestal incision and flattened using a cylindrical cutting bur under saline irrigation. Implant positions for the two preparation techniques were rotated between the two most anterior osteotomies in the P2/P3 position.

Implant osteotomies were prepared using the corresponding drills and drill sequences:

– For the standard preparation (Group 1/NP), follow the manufacturer's instructions: Round Bur ø3.1, Twist drills ø2.2, ø2.8, BL Profile Drill.
– For the overpreparation (Group 2/OP) the following drilling sequence was performed: Round Bur ø3.1, Twist drills ø2.2, ø2.8, ø3.5, BL Profile Drill.

The final drill of Group 2 resulted in an osteotomy that was wider than the maximum diameter of the implant. Osteotomies were prepared to allow implants to be placed 1 mm subcrestally. Group 2 lacked primary stability, and the implants were able to freely move in all directions to the extent that, to remove the carrier from the implant, it needed to be held in place with an instrument; therefore, no meaningful record of insertion torque was possible. Primary implant stabilities of the groups were assessed in terms of Implant Stability Quotient (ISQ). After placement, ISQ values (a.u.) were measured using the Osstell Beacon at 0° and 90°. Once all measurements had been completed, closure caps were placed NC Closure Cap H0mm, Ti for both groups. Primary closure was then achieved for submerged healing, with the mucosal margins approximated with resorbable sutures (Vicryl 4.0, Ethicon). Antibiotic cover and optional analgesia, as described above, were administered for 5 days post‐surgery (Metacam 15 mg/mL, at a dose of 0.03 mL/kg and Streptocillin 0.1 mL/kg, I.M).

### 2.6 Termination

Animals were sacrificed by intra‐cardiac injection of a 20% solution of pentobarbital (Pentobarbitalnatrium, Apoteket AB; 60 mg/mL). Block sections of the implant sites were prepared with an oscillating autopsy saw under preservation of the soft tissues and fixed in formalin (4% formaldehyde solution) for at least 2 weeks before histological processing.

### 2.7 Histological Processing

Formalin‐treated block sections were dehydrated using ascending grades of alcohol and xylene and, subsequently, infiltrated and embedded in methyl methacrylate (MMA, Sigma Aldrich; Polymerized by Perkadox 16, Nouryon) for non‐decalcified sectioning. Block sections were then cut in a buccolingual direction to sections of 500 μm (Exakts, Apparatebau, Norderstedt, Germany) (1 central section per implant) and ground to a final thickness of 30–50 μm. Sections were stained with paragon (toluidine blue and basic fuchsin) for microscopic evaluation. Histomorphometric parameters were evaluated on the buccolingual sections of each implant.

### 2.8 Histomorphometry

The primary outcome of this study was implant survival, which was defined histologically as the absence of fibrous encapsulation and the presence of direct bone‐to‐implant contact histologically.

**FIGURE 2:**
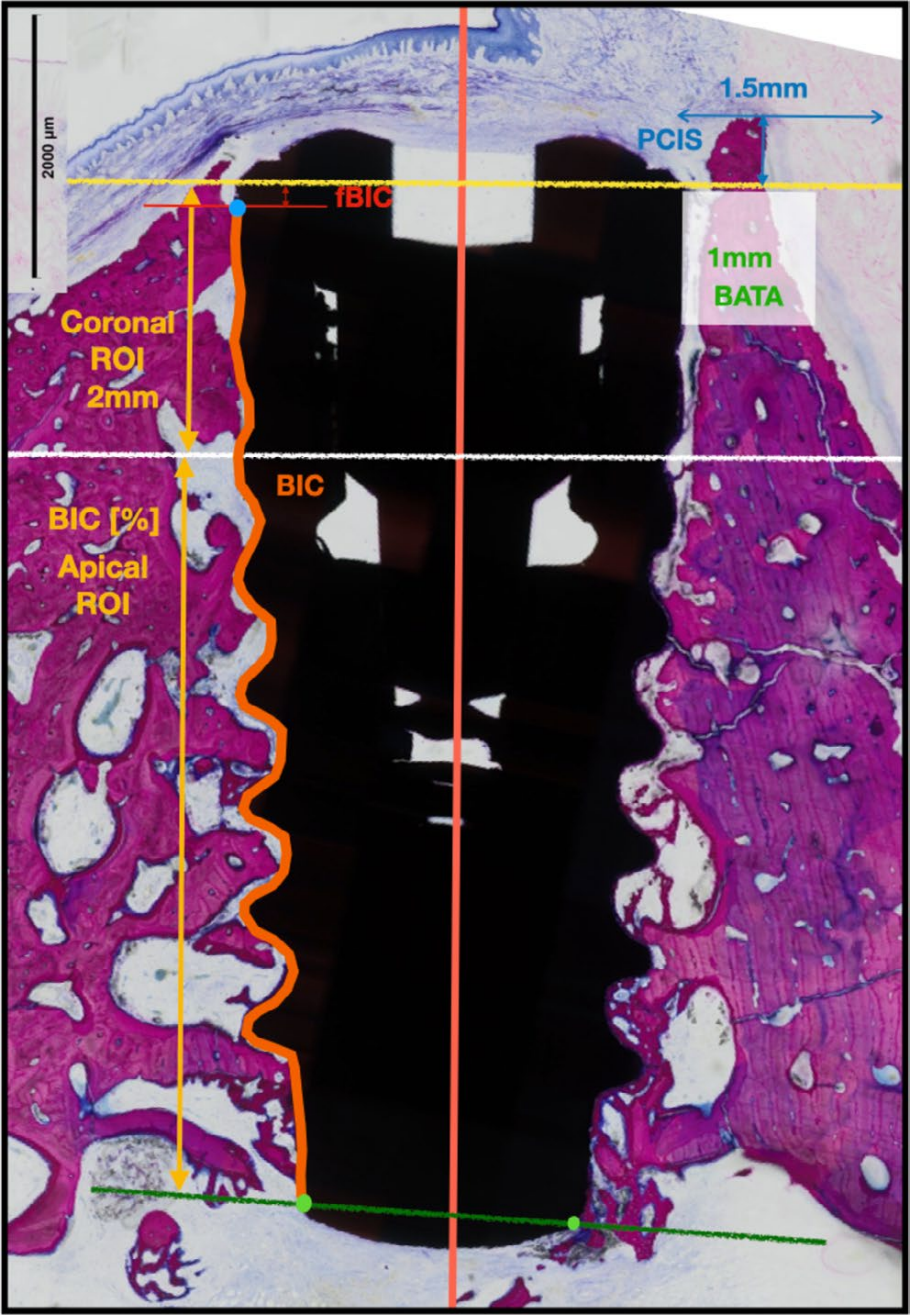
Landmarks defined for histomorphometric analysis. The orange line represents the total length for which tBIC is calculated between the first bone to implant contact and last bone to implant contact. For the Coronal ROI, cBIC is measured between the implant shoulder and 2 mm apically (white line). For the apical ROI, aBIC is measured from the white line (2 mm apical to the implant shoulder) to the last BIC (green line).

The effect of preparation type on osseointegration was determined by assessing bone‐to‐implant contact (BIC) (Figure 2). The histomorphometric parameters directly associated with this outcome were as follows:

– Total bone‐to‐implant contact (tBIC) was calculated by measuring the percentage bone‐to‐implant contact from the first bone contact to the last bone contact.
– Coronal bone‐to‐implant contact (cBIC) was calculated by measuring the percentage of bone‐to‐implant contact from the implant shoulder to 2 mm apically, thus measuring BIC in the coronal 2 mm of the implant.
– Apical bone‐to‐implant contact (aBIC) was calculated by measuring the percentage bone‐to‐implant contact from 2 mm apical to the implant shoulder to the last bone‐to‐implant contact.

Secondary outcomes related to the capacity of the individual implant surfaces to promote bone apposition and bone volume maintenance included the following:

– First bone‐to‐implant contact (fBIC) was calculated by the distance between the implant shoulder margin and the most coronal aspect of the bone in direct contact with the implant.
– Bone area to total area (BATA) was calculated by the ratio between the area occupied by bone and the total area of the region of interest. For the BATA parameter, a 1mm^2^ region of interest was assessed 1 mm adjacent and apical to the implant shoulder (See Figure 2).
– Perpendicular bone crest to implant shoulder (pCIS) as calculated by measuring the height relative to the implant shoulder of the most coronal border of bone tissue within a region of interest of 1.5 mm lateral to the implant shoulder (Francisco et al. 2021).
– pCIS bone area to total area (pCIS‐BATA) was calculated by the ratio between the area occupied by bone and total area of the region of interest. For the pCIS‐BATA region of interest, a rectangle 1 mm coronal to the implant shoulder and 1.5 mm lateral to the implant shoulder was used.

To determine the first and last bone to implant contact, 3 examiners (TG, HO, SS) independently reviewed each sample to determine their position. This was reviewed collectively, and any disagreement was discussed until a consensus was reached. Blinding of the examiners to the preparation type was not possible due to the obvious differences created by the osteotomies; however, examiners were blind to the implant surface type. Image analysis was performed using Aivia software (DRVisionTechnologies/Leica Microsystems, United Kingdom), triggering external software: python and ImageJ. First, images were loaded in Aivia (DRVisionTechnologies/Leica Microsystems) to train the Machine Learning pixel classifier to detect the implant contour and classify it as Bone‐to‐Implant Contact or non‐contact (3 classes classifier including the rest of the image as the third class). Two probability maps were output as new image channels. A second and independent pixel classifier was trained to distinguish the bone tissue from the rest of the image (2 classes classifier), creating an extra probability map as an output. Then, a python script launched from Aivia was used to manage the automated input of the raw image with the three probability maps into ImageJ for manual landmark positioning. An ImageJ macro was triggered to allow the operator to specify the position of the implant shoulder, the first bone‐to‐implant contact (fBIC), most coronal bone point for the pCIS measurement, and the last bone‐to‐implant contact (lBIC) on both sides of the implant. The coronal and apical regions were automatically computed within the same macro using the recorded positions and an orthogonal distance of 2 mm (coronal distance) from the line crossing the implant shoulders (Coronal ROI 2 mm—Figure 2).

To ensure that BIC and non‐BIC lines would have the same thickness, a combination of threshold, skeletonise and dilate operations was used in the macro. These line masks were then separated as follows using previously defined regions: left and right fBIC‐to‐lBIC, left and right coronal BIC, left and right apical BIC. To calculate the percentage of implant contact, the sum of BIC and non‐contact lines was generated for all output listed above. The ImageJ macro also generated four extra masks, extracted from automatically defined regions: left and right BATA, left and right pCIS‐BATA. The macro then exported a .csv table containing: left and right fBIC orthogonal distance to the implant shoulder, left and right pCIS orthogonal distance to the implant shoulder, all calculated from the manual landmarks positioned by the operator.

Finally, all the masks from the ImageJ macro were then processed as a batch in Aivia with the Cell Count recipe to calculate their area or length. The left and right measurements were then averaged to give a single measurement for each implant.

### 2.9 Statistical Evaluation

Values for unadjusted measured parameters were summarised as means and 95% confidence intervals. Preparation types (OP/NP) were compared using the Wilcoxon signed‐rank tests to assess for differences in primary stability as measured by ISQ. Each implant was paired within the animal to make the comparisons. A linear mixed effect model was used to assess the effect of preparation on the outcome of interest. Preparation, jaw side, tooth position and implant surface, together with the interaction between preparation and each of the other covariates, were included as fixed effects with the pig as a random effect. The assumptions of each model were checked by a study of the residuals. A secondary sub‐analysis assessed the effect of implant surface (modSLA, SLA) and preparation (OP/NP); such that a comparison within implant surface for preparation was made, for example modSLA OP and modSLA NP or SLA OP and SLA NP. Data were analysed by Stata (StataCorp. 2021. Stata Statistical Software: Release 17. College Station, TX: StataCorp LLC.) and a significance level of 0.05 was used for all hypothesis tests.

## 3 Results

### 3.1 Animal Response to Implantation and Primary Stability Assessments

All animals recovered from surgery in a predictable manner and without any intra‐r post‐surgical complications. All implants survived, resulting in a total of 60 implants in 15 animals being available for histomorphometric analysis.

At insertion, the primary stability as measured by mean ISQ for Group 1 (Normal Preparation) was 69.35 a.u. (95% CI: 68.02–70.68) and for Group 2 (Overpreparation) 11.95 a.u. (95% CI: 10.53–13.37) (Table 1). Paired comparisons demonstrated a statistically significantly lower ISQ for Group 2 compared to Group 1 (*p* < 0.001). A paired within‐animal comparison indicated that there was no evidence of a difference between the primary stability of modSLA and SLA implants placed with the same preparation type (Group 1 *p* = 0.489, Group 2 *p* = 0.142).

**TABLE 1:** Descriptive statistics for primary stability measurements as measured by mean ISQ (a.u.) with 95% CI. The column ‘All implants’ reports the mean stability measurements for all implants (*n* = 60) with the columns SLA (*n* = 30) and modSLA (*n* = 30) reporting mean stability measurements for the individual surfaces, for normal preparation (Group 1) and overpreparation (Group 2).

Preparation type	All implants; Mean ISQ (95% CI)	SLA; Mean ISQ (95% CI)	modSLA; Mean ISQ (95% CI)
Normal preparation	69.35 (68.02–70.68)	69.47 (67.46–71.47)	69.23 (67.82–70.65)
Overpreparation	11.95 (10.53–13.37)	10.7 (9.11–12.36)	13.17 (10.58–15.75)

**FIGURE 3:**
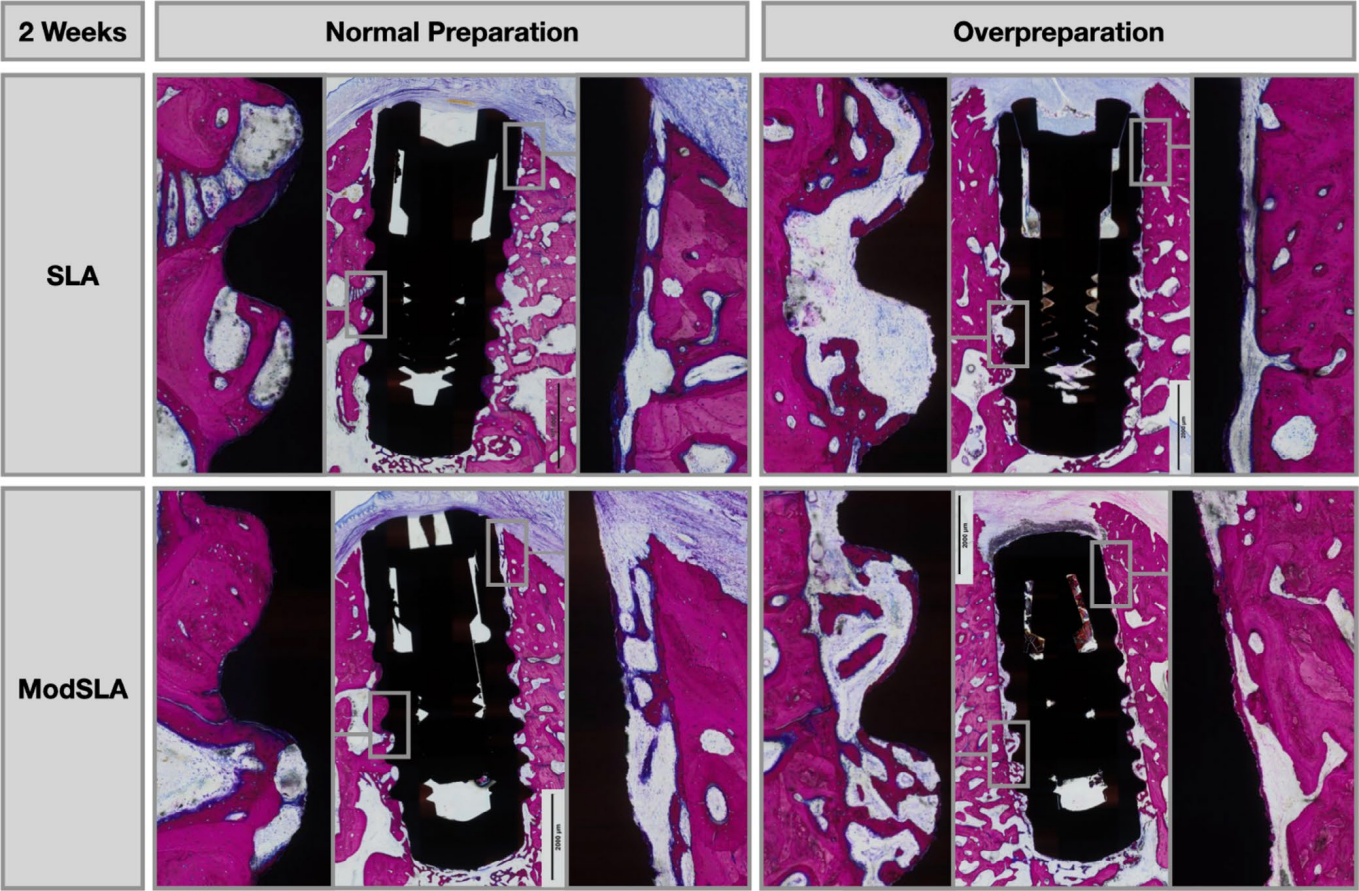
Representative micrographs at higher magnification of histological cross‐sections comparing the healing pattern of implants placed with a normal preparation (Group 1) and overpreparation (Group 2), for moderately rough modSLA implants and moderately rough SLA implants after 2 weeks of healing. Sections were stained with paragon (toluidine blue and basic fuchsin) for microscopic evaluation.

**FIGURE 4:**
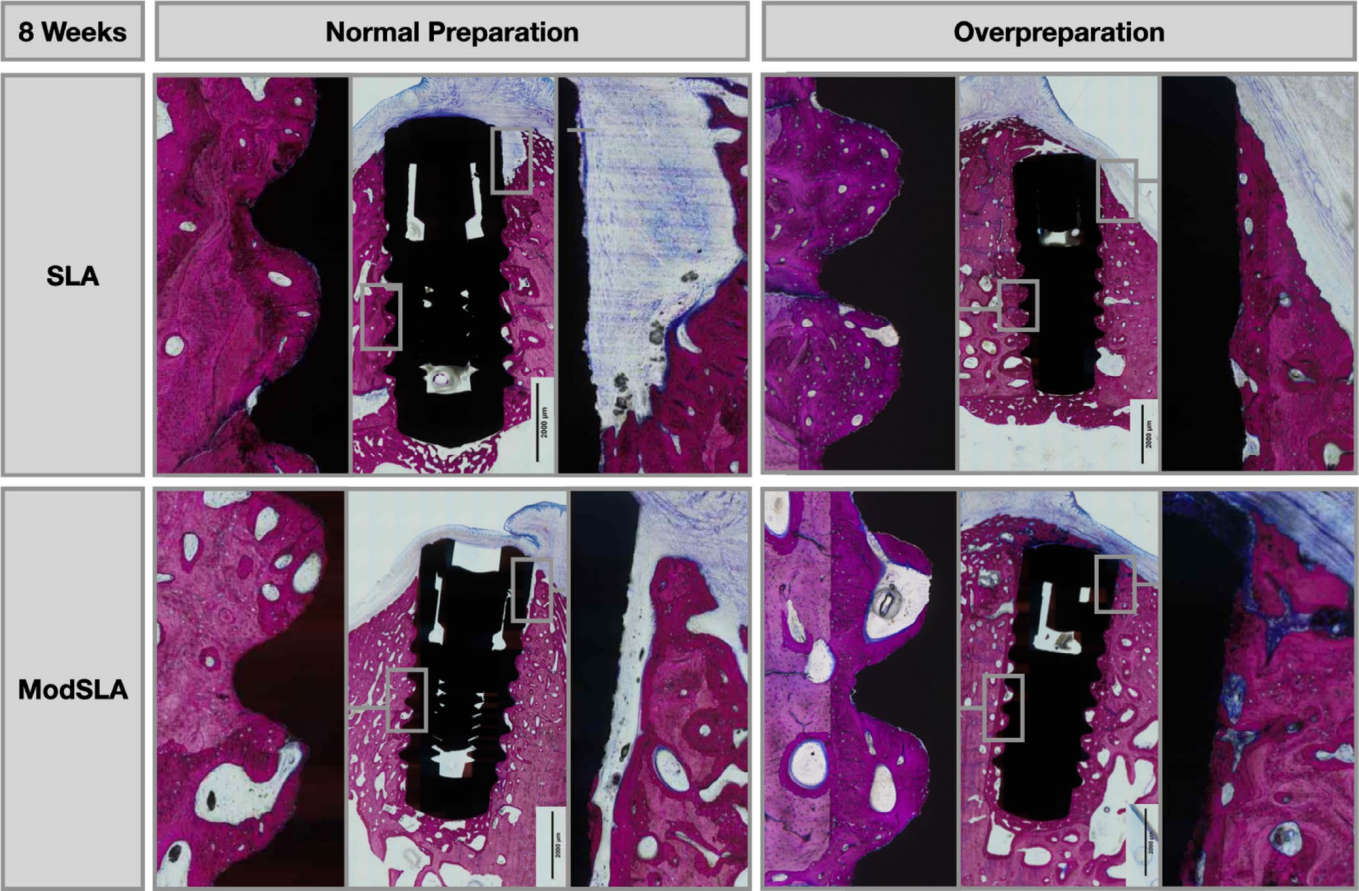
Representative micrographs at higher magnification of histological cross‐sections comparing the healing pattern of implants placed with a normal preparation (Group 1) and overpreparation (Group 2), for moderately rough modSLA implants and moderately rough SLA implants after 8 weeks of healing. Sections were stained with paragon (toluidine blue and basic fuchsin) for microscopic evaluation.

**FIGURE 5:**
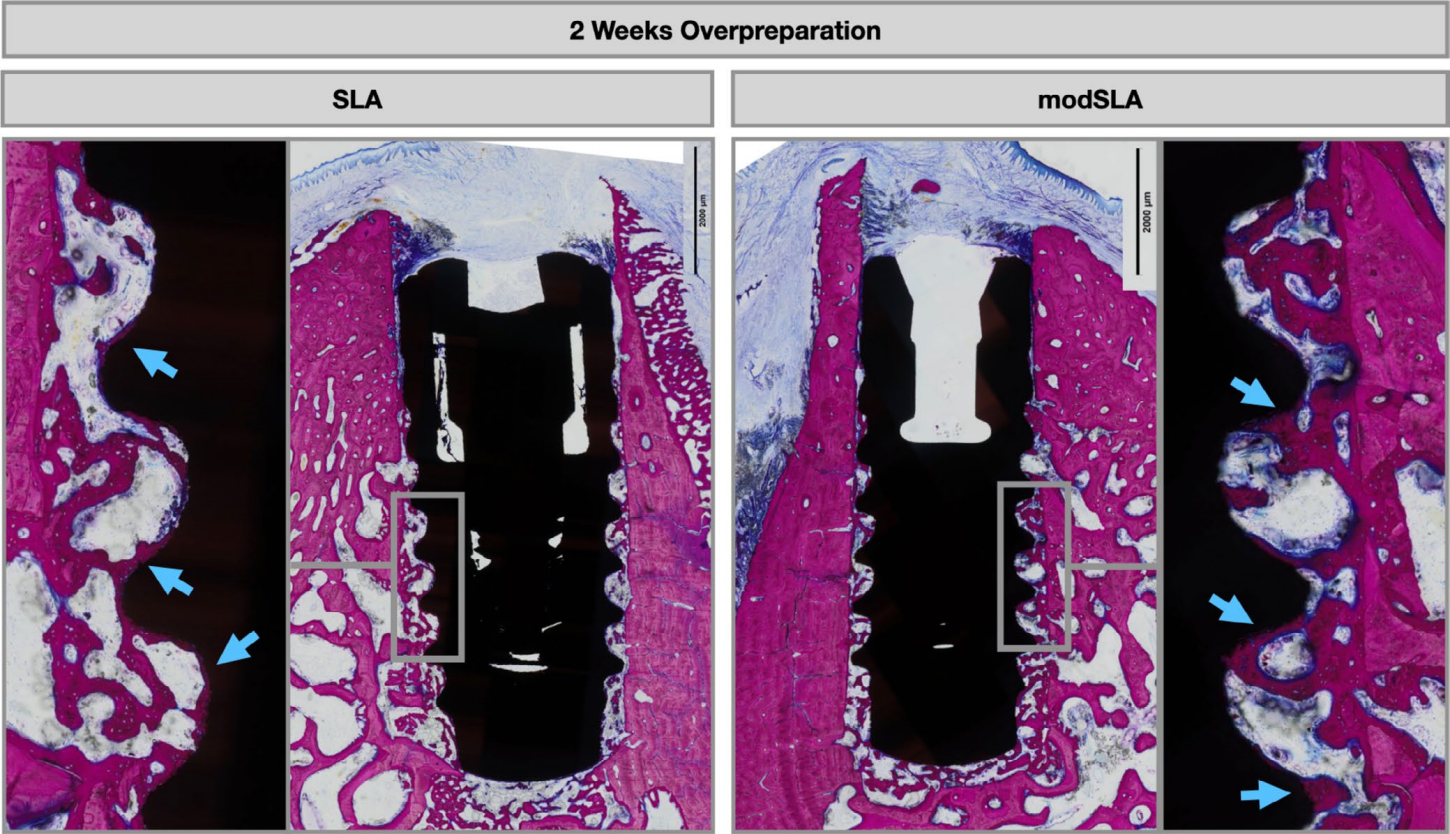
Comparison of 2 weeks healing of SLA and modSLA implants placed with an overpreparation technique (Group 2). Note the blue arrows highlight areas where de novo woven bone formation is in direct contact with the implant surface, lined with osteoblasts facing away from the implant surface.

As evidenced by the histological micrographs in Figures 3, 4, 5, distinct differences in the healing patterns for Group 1 compared to Group 2 were identified at both healing points. After 2 weeks, differences were related to the quantity of direct bone apposition to the implant surface in the coronal 2 mm (Figure 3). At the 8‐week time point, differences were mainly related to direct bone apposition in the coronal 2 mm and the height to which this contacted the implant (Figure 4).

Specifically, at 2 weeks, both SLA and modSLA in Group 2 demonstrated new de novo bone formation in contact with the implant surface (Figure 3). This newly formed woven bone was in direct contact with the implant surface, lined with osteoblasts facing away from the implant surface (Figure 5). The new bone formation for modSLA appeared thicker than that for SLA, suggesting that bone formation started earlier on the modSLA surface (Figure 5). In Group 2, at the site of osteotomy preparation or cut line, new woven bone was present through an intramembranous‐like ossification without obvious resorption.

For Group 1, bone‐to‐implant contact was commonly the result of thread engagement with the initial bone, and remodeling, resorption and apposition were common in the pitch regions.

fBIC for the SLA was more apical in Group 1 compared to Group 2, which was not the case for modSLA. At 8 weeks, new bone formation in both Groups 1 and 2 was characterised by new woven and lamellar bone (Figure 4). Observations were consistent for both surface types, with Group 2 demonstrating more coronal bone apposition and more bone volume maintained coronally around the implant shoulder.

### 3.2 Histomorphometry: Osseointegration and Crestal Bone Formation

Osseointegration in terms of BIC as a function of preparation type (Group 1/Group 2) was histomorphometrically compared after 2 and 8 weeks of healing in terms of tBIC, aBIC and cBIC. Summary values for histomorphometric outcomes are reported in Tables 2 and 3, the unadjusted values are reported in Table S1. The secondary sub‐analysis for the effect of preparation type (OP/NP) on each surface (modSLA/SLA) is reported in Table S2. The output for the linear mixed effect model is available in Table S3.

**TABLE 2:** Adjusted means and 95% confidence intervals for histomorphometric outcomes for SLA implants at 2 and 8 weeks healing timepoints.

	2 Week		8 Week	
Implant surface	SLA	SLA	SLA	SLA
Histomorphometric outcome	Normal preparation	Overpreparation	Normal preparation	Overpreparation
	Mean (95% CI)	Mean (95% CI)	Mean (95% CI)	Mean (95% CI)
fBIC (mm)	0.6 (0.43–0.77)	0.26 (0.10–0.42)	0.69 (0.55–0.82)	0.23 (0.10–0.37)
CoronalBIC (%)	31.66 (21.29–42.03)	56.76 (46.39–67.13)	35.19 (20.40–49.98)	81.97 (67.18–96.76)
ApicalBIC (%)	78.07 (71.46–84.67)	70.50 (63.89–77.10)	79.40 (73.45–85.35)	79.68 (73.73–85.63)
TotalBIC (%)	82.20 (78.26–86.12)	70.93 (66.99–74.87)	84.63 (80.36–88.90)	77.09 (72.82–81.37)
PCIS (mm)	0.11 (0.03–0.18)	0.28 (0.20–0.35)	0.46 (0.36–0.57)	0.28 (0.18–0.39)
PCIS BATA (%)	9.78 (−1.81–21.36)	50.16 (38.58–61.74)	15.23 (5.88–24.59)	26.97 (17.61–36.32)
BATA (%)	50.53 (41.44–59.63)	70.7 (61.67–79.86)	34.30 (21.14–47.46)	59.17 (46.01–72.33)

**TABLE 3:** Adjusted means and 95% confidence intervals for histomorphometric outcomes for modSLA implants at 2 and 8 weeks healing timepoints.

	2 Week		8 Week	
Implant surface	modSLA	modSLA	modSLA	modSLA
Histomorphometric outcome	Normal preparation	Overpreparation	Normal preparation	Overpreparation
	Mean (95% CI)	Mean (95% CI)	Mean (95% CI)	Mean (95% CI)
fBIC (mm)	0.51 (0.35–0.67)	0.54 (0.38–0.70)	0.37 (0.24–0.50)	0.11 (−0.03–0.24)
CoronalBIC (%)	28.88 (18.51–39.25)	39.25 (28.89–49.63)	50.74 (35.95–65.52)	76.76 (61.97–91.55)
ApicalBIC (%)	74.90 (68.30–81.50)	54.68 (48.08–61.28)	78.57 (72.62–84.52)	69.09 (63.14–75.04)
TotalBIC (%)	68.70 (64.77–72.64)	55.97 (52.04–59.91)	74.09 (69.81–78.36)	66.55 (62–28—70.82)
PCIS (mm)	0.15 (0.08–0.22)	0.22 (0.15–0.29)	0.25 (0.14–0.35)	0.12 (0.01–0.22)
PCIS BATA (%)	9.61 (−1.97–21.20)	26.19 (14.51–37.78)	9.79 (0.43–19.14)	9.62 (0.26–18.97)
BATA (%)	32.19 (23.10–41.29)	54.43 (45.34–63.52)	27.20 (14.04–40.36)	48.48 (35.33–61.64)

Osseointegration as measured by tBIC demonstrated no significant difference in the mean tBIC between Groups 1 and 2 at both 2 and 8 weeks in the mixed effects linear regression analysis (2 W *p* = 0.305, 8 W *p* = 0.423) (Table 4). At 8 weeks, the mean tBIC ranged between 79.15% and 82.78% for the two preparation types. There was no evidence of a difference for the aBIC outcome between Group 1 and Group 2 at 2 or 8 weeks (2 W *p* = 0.858, 8 W *p* = 0.716) (Table 4), with both preparation types at 8 weeks having similar mean BICs (Group 1 mean = 78.99%, Group 2 mean 74.38%). At 2 and 8 weeks, the ModSLA surface demonstrated significantly lower aBIC for Group 2 (2 W Difference in means = −21.29, 95% CI: −30.74 to −11.84, *p* < 0.001, 8 W Difference in means = −9.42, 95% CI: −16.26 to −2.57, *p* = 0.007). At 8 weeks, mean aBIC for modSLA implants in Group 1 was 78.57% (95% CI: 2.62–84.52), compared to 69.09% (95% CI: 63.14–75.04) for Group 2.

**TABLE 4:** A comparison of histomorphometric outcomes between overpreparation (Group 2)and normal preparation (Group 1). Mixed linear models (adjusted for the fixed effects of mandible, tooth position and implant surface with the individual test animal as a random effect) were used to estimate the effects of interest.

Healing time (weeks)	Histomorphometric outcome	Mixed‐effect linear regression^a^			
		Coefficient^b^	95% CI for adjusted mean difference		*p* *
2	fBIC (mm)	−0.53	−0.84	−0.23	0.001
	CoronalBIC (%)	41.29	21.25	61.33	< 0.001
	ApicalBIC (%)	−1.18	−14.05	11.69	0.858
	TotalBIC (%)	−4.01	11.69	3.66	0.305
	PCIS (mm)	0.20	0.06	0.34	0.005
	PCIS BATA (%)	51.70	29.12	74.28	< 0.001
	BATA (%)	35.30	18.09	522.51	< 0.001
8	fBIC (mm)	−0.52	−0.79	−0.26	< 0.001
	CoronalBIC (%)	42.86	13.04	72.68	0.005
	ApicalBIC (%)	2.13	−9.35	13.60	0.716
	TotalBIC (%)	−4.58	−15.79	6.62	0.423
	PCIS (mm)	−0.31	−0.52	−0.10	0.004
	PCIS BATA (%)	17.78	−1.08	36.64	0.065
	BATA (%)	28.54	2.01	55.08	0.035

^a^ The effect of the animal was introduced as a random effect.

^b^ Each coefficient represents the estimated difference in means of the relevant outcome between overpreparation compared to normal preparation (a positive value implies that the mean for OP > mean for NP).

* *p*‐value adjusted for multiple comparisons using the Dunnett‐Hsu Test method.

At 2 weeks, osseointegration in the coronal 2 mm of the implants (cBIC) demonstrated significantly higher mean levels of bone apposition for Group 2 (*p* < 0.001). On average, Group 2 demonstrated 41.3% more cBIC than Group 1 (Difference in means = 41.29%, 95% CI: 21.25–61.33, *p* < 0.001, Table 4). This relationship was maintained at 8 weeks (Difference in means = 42.86%, 95% CI: 13.04–72.68, *p* = 0.005, Table 4). The mean cBIC for Group 2 at 8 W was 79.37% (95% CI: 67.99–90.74) compared to 42.96% (95% CI: 29.82–56.11) for Group 1 NP.

At 2 weeks, only the SLA surface in Group 2 demonstrated a higher % cBIC compared to Group 1 (Difference in means = 24.56, 95% CI: 11.19–37.92, *p* < 0.001). There was evidence of a difference for the ModSLA surface (Difference in means = 10.92%, 95% CI: −2.44 to −24.29, *p* = 0.109) (Table S2). At 8 weeks, both SLA and ModSLA demonstrated a significantly higher mean cBIC for Group 2 compared to Group 1 (SLA Difference in means = 46.78, 95% CI: 25.86–67.69, *p* < 0.001, ModSLA Difference in means = 26.44, 95% CI: 5.18–47.69, *p* = 0.015).

Crestal bone volume maintenance in terms of percentage of bone as a function of total volume was assessed histomorphometrically after 2 and 8 weeks in terms of BATA and pCIS‐BATA. BATA in the first 1mm^2^ of the implant coronally was significantly higher at both 2 and 8 weeks for Group 2 compared to Group 1 (2w Difference in means = 35.30%, 95% CI: 18.09–52.51, *p* < 0.001) (8w Difference in means = 28.54%, 95% CI: 2.01–55.08, *p* = 0.035). For both SLA and ModSLA surfaces at 2‐ and 8‐week timepoints, Group 2 resulted in significantly higher BATA compared to Group 1 (2w SLA *p* < 0.001, modSLA *p* < 0.001, 8w SLA *p* = 0.009, modSLA *p* = 0.028, Table S2). At 8 weeks, Group 2 maintained 53.83% (95% CI: 40.58–67.07) of bone around the implant shoulder compared to 30.75% (95% CI: 21.47–40.03) for Group 1.

At 2 weeks, there was significantly higher pCIS‐BATA for Group 2 compared to Group 1 (Difference in means = 51.70%, 95% CI: 29.12–74.28, *p* < 0.001). Moreover, both SLA and ModSLA demonstrated significantly higher pCIS‐BATA for Group 2 (SLA *p* < 0.001, modSLA *p* = 0.043). However, at 8 weeks, there was no difference between the two groups (Difference in means = 17.78%, 95% CI: −1.08 to −36.64, *p* = 0.065) or for surface types (SLA *p* = 0.082, ModSLA *p* = 0.948, Table S2).

The height to which new bone formed was compared between groups after 2 and 8 weeks of healing and was assessed by fBIC. A positive value represents a more apical position, whereas a negative value represents a more coronal position. At 2 weeks, Group 2 demonstrated a significantly more coronal fBIC than Group 1 (Coefficient: −0.53 mm, 95% CI: −0.84 to −0.23, *p* = 0.001). Similarly, at 8 weeks, Group 2 demonstrated significantly more coronal fBIC (Coefficient: = −0.52 mm, 95% CI: −0.79 to −0.26, *p* = 0.009). On average, Group 2 resulted in a 0.5 mm more coronal fBIC for both timepoints compared to Group 1. At 8 weeks, the average fBIC for SLA and modSLA implants was 0.23 mm (0.10–0.37) and 0.11 mm (0.03–0.24), respectively, from the implant shoulder.

At 2 weeks, SLA in Group 2 resulted in more coronal bone apposition (*p* < 0.001, Table S2). In contrast, there was no evidence of a difference in fBIC between preparation types for the ModSLA (*p* = 0.610, Table S2). However, at 8 weeks both surfaces (SLA and ModSLA) for Group 2 demonstrated a more coronal fBIC (SLA *p* < 0.001, modSLA *p* = 0.003).

Maintenance of crestal bone levels was evaluated in terms of PCIS, where a positive value represented the highest bone peak above the implant shoulder, and a negative value represented bone positioned below the implant shoulder. At both 2‐and 8‐week timepoints, Group 2 demonstrated significantly more coronal bone compared to Group 1 (2w Coefficient = 0.42 mm, 95% CI: 0.22–0.62, *p* ≤ 0.001, 8w Coefficient = 0.65 mm, 95% CI: 0.39–0.91, *p* < 0.001). At 2 weeks, this relationship was maintained for both surface types (SLA *p* < 0.001, ModSLA *p* = 0.026). However, at 8 weeks, only the SLA surface demonstrated significantly more coronal bone levels for Group 2 compared to Group 1 (*p* < 0.001). In contrast, there was no evidence of a difference between preparation types for the ModSLA surface at 8 weeks (*p* = 0.106, Table S2).

## 4 Discussion

This study investigated the influence of an oversized osteotomy preparation that resulted in a complete lack of primary stability on osseointegration. The impact of preparation type and thus primary stability on osseointegration was examined by comparing a standard preparation as per the manufacturer's instructions (Group 1) with an overprepared osteotomy (Group 2) that resulted in an osteotomy wider than the maximum diameter of the implant and permitted macro‐movement of the implant within the osteotomy.

From the comparison of the study groups, the following salient observations were made: (A) All implants placed in Group 2, with an average ISQ of 12 a.u., successfully osseointegrated. (B) Group 2 resulted in significantly higher fBIC, BATA and cBIC compared to the standard preparation of Group 1. (C) Qualitative assessment suggested higher early de novo bone formation at the implant‐bone interface for the modSLA surface compared to SLA surface implants at 2 weeks. (D) At 2 and 8 weeks, there was no difference in the percentage of bone‐to‐implant contact as measured from the fBIC to the last‐BIC (tBIC) and from 2 mm below the shoulder to the last‐BIC (aBIC) between the preparation types.

This study found that implants placed with no primary stability successfully osseointegrated. Within the limits of this study, no difference was observed in implant survival between implants with low primary stability and those placed conventionally. This supports the clinical studies reporting high implant success rates for low primary stability measurements in non‐immediately loaded cases (Norton 2017; Berardini et al. 2016; Razaghi et al. 2025). In this study, submerged implants in overprepared osteotomies that could be readily displaced with a very light force predictably osseointegrated. Implant displacement or macromotion during healing continues to influence clinical practice and is considered to be deleterious for osseointegration, with fibrous tissue encapsulation occurring (Szmukler‐Moncler et al. 1998; Lioubavina‐Hack et al. 2006; Trisi et al. 2009). Implant micromotion is a relevant mechanobiological mechanism that may also positively drive osseointegration (Simmons et al. 2001; Wazen et al. 2013; Rubin and McLeod 1994; Vandamme et al. 2007). However, the tolerable threshold for implant micromotion is complex, with large ranges of acceptable micromotion being reported in pre‐clinical studies (Kohli et al. 2021). Pre‐clinical studies support the conclusion that submerged and unloaded implants can result in successful osseointegration, even if mechanical engagement at placement was not obtained (Ivanoff et al. 1996; Duyck et al. 2015; Jung et al. 2012; Rea et al. 2015; Yurttutan et al. 2016; Cohen et al. 2016; Huang et al. 2020). Although not statistically significant, overpreparation in this study resulted in lower BIC in the Apical ROI than normal preparation. A previous preclinical study that utilised an end timepoint of 4 months with submerged unloaded healing found no difference in BIC between a conventional preparation (IT ≈30), an underprepared osteotomy by 0.3 mm (IT > 70) and an overprepared osteotomy of 0.2 mm (IT ≈0) (Rea et al. 2015). At 2 weeks, this study demonstrated comparably higher BIC measurements for overpreparation compared to previous studies by Duyck et al. (2015) and Huang et al. (2020). However, this may be the result of differences in the animal models utilised, as these studies were conducted in rabbit tibiae.

Preventing implant micromotion does not assure robust osseointegration (Sivolella et al. 2012). Stable implants placed in oversized osteotomies without primary bone contact demonstrated significantly lower BIC (5.3%) after 3 months compared to stabilised implants which maintained bone contact (46.1%) (Sivolella et al. 2012). Therefore, it seems that the relationship between osseointegration and primary stability is not a simple dichotomy but a much more complex interplay of implant‐bone loading and biological response.

In this study, high BIC was demonstrated for both SLA and modSLA. Implant surface characteristics may affect osseointegration in the absence of primary stability (Jung et al. 2014). At earlier time points, modSLA implants have demonstrated higher BIC than SLA implants (Buser et al. 2004; Bornstein et al. 2008; Schwarz, Ferrari, et al. 2007). However, after 8 weeks of healing, similar bone apposition outcomes between SLA and modSLA have been reported (Lai et al. 2009; Liñares et al. 2011). Submerged healing and osseointegration in pre‐clinical models have previously been well‐characterised (Caroprese et al. 2017; Berglundh et al. 2003; Rossi et al. 2014). Implants that achieved primary stability with circumferential defects demonstrate bone formation without signs of prior resorption and appositional growth from the lateral and apical bone walls of the defect. This osseous growth pattern displays a similar osteoblast arrangement along the implant surface as seen in this study, for an intramembranous‐like ossification (Botticelli et al. 2003). It is hypothesised that these sites are filled with a blood clot immediately after placement, allowing the rapid formation of woven bone (Berglundh et al. 2003). Osseointegration of circumferential gap‐defects up to 1.25mmm have been shown to regenerate predictably (Berglundh et al. 2003; Lai et al. 2009; Campos et al. 2012; Rossi et al. 2012). Notwithstanding, the permissible gap for spontaneous bone infill remains unknown (Lai et al. 2009).

The effect of overpreparation on bone‐implant contact seems to act independently of roughness, with the same relationship for machined titanium implants that are non‐stable showing higher bone‐to‐implant and bone volume maintenance compared with stable implants after 4 weeks of healing (Buser et al. 1991; Meirelles et al. 2007). Interestingly, this study noted a less pronounced difference in the effect of overpreparation for the modSLA surface compared to standard preparation on coronal bone apposition as measured by fBIC. This suggests that the super‐hydrophilic surface was able to reduce the negative effect of the standard preparation compared to overpreparation on coronal bone apposition. This may be due to the enhanced ability of modSLA implants to stabilise the blood clot, allowing the formation of a well‐organised preliminary collagen‐rich matrix to support coronal bone apposition (Schwarz, Ferrari, et al. 2007). Interestingly, at the 2‐week time point, qualitatively, new bone formation at the modSLA surface appeared thicker compared to the SLA surface, indicating an earlier initiation of bone formation. However, on average, BIC was higher for the SLA surface in this study for both preparation types, although no direct comparison between the surfaces was made. This finding contrasts with previously published studies that have consistently reported higher BIC measurements for hydrophilic surfaces (Schwarz, Herten, et al. 2007; Shahdad et al. 2025). It is possible under the idealised conditions of this study, where the implants are surrounded fully by bone, stabilisation of the blood clot is less critical for BIC.

This study demonstrated comparably higher levels of crestal bone loss for implants placed with high primary stability using a standard osteotomy preparation compared to an overprepared osteotomy with absent primary stability (Jung et al. 2012). Although high insertion torques have not been shown to prevent integration, studies have reported higher levels of peri‐implant bone remodeling and buccal soft tissue recession with implants placed under higher insertion torques (Khayat et al. 2013; Barone et al. 2016; Aldahlawi et al. 2018). High insertion torques can cause an increase in the inflammatory response and resorption of crestal bone around implants (Gehrke et al. 2022) due to pressure necrosis. When considering the results of this study, decreasing the compression of the bone around the crest may avoid undesirable resorption (Eom et al. 2016; Marin et al. 2016). A similar observation has been reported clinically (Seleem et al. 2021), highlighting the importance of optimizing surgical protocols to mitigate the risk of adverse early marginal bone remodeling by reducing the compression forces transmitted to the surrounding bone during implant insertion.

This study is the first study to examine osseointegration of non‐stable implants utilizing the mandibular minipig model, examining how in both super‐hydrophilic moderately rough sandblasted acid etched surfaces (modSLA) and its relatively hydrophobic version (SLA) osseointegrate. The minipig model has been demonstrated to be an appropriate in vivo model to study the osseointegration process and alveolar remodeling, having similar anatomical and healing characteristics to human bone (Musskopf et al. 2022). This study demonstrated similar osseointegration to that observed in other preclinical animal models (Cohen et al. 2016; Rea et al. 2015; Huang et al. 2020). Therefore, osseointegration in humans may develop similarly in a clinical setting. In this study, implants were not functionally loaded, and although a submerged healing protocol was utilised, some loading during healing could not be excluded due to masticatory forces. BIC has been positively correlated with removal torque, and therefore, higher BIC can infer improved osseointegration (Johansson and Albrektsson 1987; Cho and Park 2003). Previous studies with comparable BIC measurements have reported high removal torques for modSLA and SLA implants after up to 8 weeks of healing (Ferguson et al. 2006; Abdel‐Haq et al. 2011). Therefore, implants in our study would likely be suitable for functional loading.

Preclinical studies, including ours, that assess overpreparation have predominantly been conducted under optimal ridge width conditions during submerged healing. Thus, extending these findings to other clinical scenarios that involve concurrent bone defects requires further investigation. Nonetheless, based on the authors clinical experience, non‐immediately loaded implants placed without primary stability, even in the presence of a dehiscence or fenestration defect, have consistently achieved healing and osseointegration.

In light of the existing body of preclinical and clinical studies, which have demonstrated successful osseointegration even with low or absent primary stability, conventional views such as “primary stability is a prerequisite for successful osseointegration” (Lioubavina‐Hack et al. 2006) should be reevaluated. A more accurate assertion might be that it is crucial to ‘avoid excessive micromotion during healing’ rather than to ensure primary stability. Additionally, the optimal distribution of pressure by implant threads, especially avoiding significant pressure at the coronal aspect of the implant, is critical for maintaining crestal bone levels. Future studies should further investigate these aspects.

## 5 Conclusion

This study demonstrated that in a porcine preclinical model, implants placed with a submerged healing, without primary stability, osseointegrate. Further, overpreparation resulted in comparable bone‐toto‐implant contact to standard implant preparation. However, when considering the coronal 2 mm, overpreparation resulted in significantly more coronal bone‐to‐implant contact and bone volume maintenance around the implant shoulder.

## Author Contributions

**Thomas Gill:** methodology, validation, writing – review and editing, writing – original draft, data curation, investigation, resources, formal analysis, visualization. **Hansley Ooi:** investigation, methodology, writing – review and editing, data curation, resources. **Emre Tezulas:** data curation, resources, investigation, writing – review and editing, methodology. **Aviva Petrie:** writing – review and editing, investigation, formal analysis, software, data curation. **Simon Rawlinson:** writing – review and editing, formal analysis, data curation, supervision, investigation. **Mario Roccuzzo:** conceptualization, investigation, funding acquisition, writing – review and editing, resources, formal analysis, methodology. **Shakeel Shahdad:** conceptualization, investigation, funding acquisition, writing – review and editing, methodology, supervision, resources, formal analysis.

## Ethics Statement

This article does not contain any studies with human participants performed by any of the authors. Ethical standards related to the research of human subjects are not applicable. All applicable international, national and/or institutional guidelines for the care and use of animals were followed.

## Consent

The authors have nothing to report.

## Conflicts of Interest

The present study was funded by a grant from Institut Straumann AG. S.S. and M.R. receive speaker honorariums for educational courses from Institut Straumann AG. T.G., H.O., E.T., A.P. and S.R. declare no conflicts of interest.

## Supporting information


**Table S1.** Unadjusted means and 95% confidence intervals for histomorphometric outcomes at 2 and 8 weeks healing timepoints.
**Table S2.** Linear mixed effect model, secondary sub‐analysis to assess the effect of implant surface (modSLA, SLA) and preparation (OP/NP).
**Table S3.** Linear mixed effect model.

## Data Availability

The data that support the findings of this study are available from the corresponding author upon reasonable request.
