# Supplementary material for: Osseointegration in the Absence of Primary Stability: An Experimental Preclinical Mandibular Minipig Overpreparation In Vivo Model
Source: Clin Oral Implants Res. 2025 Jul 23;36(10):1346–59. doi: 10.1111/clr.70006 (PMC12491923; doi:10.1111/clr.70006)
Supplement: Supplementary file 1 — Table S1. Unadjusted means and 95% confidence intervals for histomorphometric outcomes at 2 and 8 weeks healing timepoints. Table S2. Linear mixed effect model, secondary sub‐analysis to assess the effect of implant surface (modSLA, SLA) and preparation (OP/NP). Table S3. Linear mixed effect model. [file CLR-36-1346-s001.docx]

**Supplementary Tables**

**Supplementary Table 1:** Unadjusted means and 95% confidence intervals for histomorphometric outcomes at 2 and 8 weeks healing timepoints.

|  | 2 Weeks | | 8 Weeks | |
| --- | --- | --- | --- | --- |
| Histomorphometric Outcome | Normal Preparation | Overpreparation | Normal Preparation | Overpreparation |
|  | Mean (95%CI) | Mean (95%CI) | Mean (95%CI) | Mean (95%CI) |
| fBIC (mm) | 0.56 (0.45 – 0.67) | 0.40 (0.21 – 0.59) | 0.53 (0.36 – 0.69) | 0.17 (0.10 – 0.15) |
| CoronalBIC (%) | 30.27 (22.33 - 38.21) | 48.01 (36.50 - 59.52) | 42.96 (29.82 - 56.11) | 79.37 (67.99 - 90.74) |
| ApicalBIC (%) | 76.48 (70.13 - 82.84) | 62.59 (55.18 - 69.99) | 78.99 (74.45 - 83.53) | 74.38 (68.52 - 80.25) |
| TotalBIC (%) | 75.45 (70.64 - 80.26) | 63.45 (56.56 - 70.34) | 82.78 (79.24 - 86.32) | 79.15 (73.02 - 85.27) |
| pCIS (mm) | 0.04 (-0.03 – 0.11) | 0.24 (0.13 – 0.36) | -0.28 (-0.43 - -0.13) | 0.01 (0.09 – 0.11) |
| pCIS BATA (%) | 9.69 (6.08 - 13.30) | 38.18 (22.99 - 53.37) | 12.51 (6.62 - 18.41) | 18.29 (7.83 - 28.75) |
| BATA (%) | 41.36 (30.64 - 52.09) | 62.60 (52.83 - 72.36) | 30.75 (21.47 - 40.03) | 53.83 (40.58 - 67.07) |

**Supplementary Table 2:** Linear Mixed Effect Model, secondary sub-analysis to assess the effect of implant surface (modSLA,SLA) and preparation (OP/NP);

| **Outcome** | fBIC | **Timepoint** | 2 weeks |  |  |  |
| --- | --- | --- | --- | --- | --- | --- |
|  | dy/dx | Delta-method | z | P>\|z\| | 95% conf. interval | |
|  |  | std. err. |  |  |  |  |
| 0. Preparation | (base outcome) | | | | | |
| 1. Preperation |  | | | | | |
| Implant Surface |  |  |  |  |  |  |
| SLA | -368.744 | 101.082 | -3.650 | 0.000 | 566.861 | -170.627 |
| modSLA | 51.509 | 101.082 | 0.510 | 0.610 | -146.608 | 249.627 |
|  |  |  |  |  |  |  |
|  |  |  |  |  |  |  |
| **Outcome** | fBIC | **Timepoint** | 8 weeks |  |  |  |
|  | dy/dx | Delta-method | z | P>\|z\| | 95% conf. interval | |
|  |  | std. err. |  |  |  |  |
| 0. Preparation | (base outcome) | | | | | |
| 1. Preperation |  | | | | | |
| Implant Surface |  |  |  |  |  |  |
| SLA | -450.466 | 86.462 | -5.210 | 0.000 | -619.928 | -281.004 |
| modSLA | -258.664 | 87.868 | -2.940 | 0.003 | -430.882 | -86.446 |
|  |  |  |  |  |  |  |
|  |  |  |  |  |  |  |
| **Outcome** | pCIS | **Timepoint** | 2 Weeks |  |  |  |
|  | dy/dx | Delta-method | z | P>\|z\| | 95% conf. interval | |
|  |  | std. err. |  |  |  |  |
| 0. Preparation | (base outcome) | | | | | |
| 1. Preperation |  | | | | | |
| Implant Surface |  |  |  |  |  |  |
| SLA | 166.033 | 53.271 | 3.120 | 0.002 | 61.625 | 270.442 |
| modSLA | 70.874 | 53.271 | 1.330 | 0.193 | -33.535 | 175.283 |
|  |  |  |  |  |  |  |
|  |  |  |  |  |  |  |
| **Outcome** | pCIS | **Timepoint** | 8 Weeks |  |  |  |
|  | dy/dx | Delta-method | z | P>\|z\| | 95% conf. interval | |
|  |  | std. err. |  |  |  |  |
| 0. Preparation | (base outcome) | | | | | |
| 1. Preperation |  | | | | | |
| Implant Surface |  |  |  |  |  |  |
| SLA | -178.158 | 69.247 | -2.570 | 0.010 | -313.879 | -42.438 |
| modSLA | -118.231 | 70.373 | -1.680 | 0.093 | -256.159 | 19.697 |
|  |  |  |  |  |  |  |
|  |  |  |  |  |  |  |
| **Outcome** | tBIC | **Timepoint** | 2 Weeks |  |  |  |
|  | dy/dx | Delta-method | z | P>\|z\| | 95% conf. interval | |
|  |  | std. err. |  |  |  |  |
| 0. Preparation | (base outcome) | | | | | |
| 1. Preperation |  | | | | | |
| Implant Surface |  |  |  |  |  |  |
| SLA | -10.457 | 2.875 | -3.640 | 0.000 | -16.093 | -4.822 |
| modSLA | -13.540 | 2.875 | -4.710 | 0.000 | -19.175 | -7.904 |
|  |  |  |  |  |  |  |
|  |  |  |  |  |  |  |
| **Outcome** | tBIC | **Timepoint** | 8 Weeks |  |  |  |
|  | dy/dx | Delta-method | z | P>\|z\| | 95% conf. interval | |
|  |  | std. err. |  |  |  |  |
| 0. Preparation | (base outcome) | | | | | |
| 1. Preperation |  | | | | | |
| Implant Surface |  |  |  |  |  |  |
| SLA | -4.050 | 3.737 | -1.080 | 0.278 | 11.375 | 3.275 |
| modSLA | -2.343 | 3.798 | -0.620 | 0.537 | -9.787 | 5.101 |
|  |  |  |  |  |  |  |
|  |  |  |  |  |  |  |
| **Outcome** | cBIC | **Timepoint** | 2 Weeks |  |  |  |
|  | dy/dx | Delta-method | z | P>\|z\| | 95% conf. interval |  |
|  |  | std. err. |  |  |  |  |
| 0. Preparation | (base outcome) | | | | | |
| 1. Preperation |  | | | | | |
| Implant Surface |  |  |  |  |  |  |
| SLA | 24.556 | 6.818 | 3.600 | 0.000 | 11.192 | 37.920 |
| modSLA | 10.922 | 6.818 | 1.600 | 0.109 | -2.442 | 24.286 |
|  |  |  |  |  |  |  |
|  |  |  |  |  |  |  |
| **Outcome** | cBIC | **Timepoint** | 8 Weeks |  |  |  |
|  | dy/dx | Delta-method | z | P>\|z\| | 95% conf. interval |  |
|  |  | std. err. |  |  |  |  |
| 0. Preparation | (base outcome) | | | | | |
| 1. Preperation |  | | | | | |
| Implant Surface |  |  |  |  |  |  |
| SLA | 46.778 | 10.671 | 4.380 | 0.000 | 25.863 | 67.693 |
| modSLA | 26.435 | 10.845 | 2.440 | 0.015 | 5.180 | 47.690 |
|  |  |  |  |  |  |  |
|  |  |  |  |  |  |  |
| **Outcome** | aBIC | **Timepoint** | 2 Weeks |  |  |  |
|  | dy/dx | Delta-method | z | P>\|z\| | 95% conf. interval |  |
|  |  | std. err. |  |  |  |  |
| 0. Preparation | (base outcome) | | | | | |
| 1. Preperation |  | | | | | |
| Implant Surface |  |  |  |  |  |  |
| SLA | -6.497 | 4.823 | -1.350 | 0.178 | -15.950 | 2.956 |
| modSLA | -21.292 | 4.823 | -4.410 | 0.000 | -30.745 | -11.839 |
|  |  |  |  |  |  |  |
|  |  |  |  |  |  |  |
| **Outcome** | aBIC | **Timepoint** | 8 Weeks |  |  |  |
|  | dy/dx | Delta-method | z | P>\|z\| | 95% conf. interval |  |
|  |  | std. err. |  |  |  |  |
| 0. Preparation | (base outcome) | | | | | |
| 1. Preperation |  | | | | | |
| Implant Surface |  |  |  |  |  |  |
| SLA | 0.282 | 3.436 | 0.080 | 0.935 | -6.453 | 7.017 |
| modSLA | -9.418 | 3.492 | -2.700 | 0.007 | 16.263 | -2.573 |
|  |  |  |  |  |  |  |
|  |  |  |  |  |  |  |
| **Outcome** | BATA | **Timepoint** | 2 Weeks |  |  |  |
|  | dy/dx | Delta-method | z | P>\|z\| | 95% conf. interval |  |
|  |  | std. err. |  |  |  |  |
| 0. Preparation | (base outcome) | | | | | |
| 1. Preperation |  | | | | | |
| Implant Surface |  |  |  |  |  |  |
| SLA | 20.484 | 5.422 | 3.780 | 0.000 | 9.856 | 31.111 |
| modSLA | 21.984 | 5.422 | 4.050 | 0.000 | 11.357 | 32.612 |
|  |  |  |  |  |  |  |
|  |  |  |  |  |  |  |
| **Outcome** | BATA | **Timepoint** | 8 Weeks |  |  |  |
|  | dy/dx | Delta-method | z | P>\|z\| | 95% conf. interval |  |
|  |  | std. err. |  |  |  |  |
| 0. Preparation | (base outcome) | | | | | |
| 1. Preperation |  | | | | | |
| Implant Surface |  |  |  |  |  |  |
| SLA | 24.865 | 9.495 | 2.620 | 0.009 | 6.256 | 43.475 |
| modSLA | 21.225 | 9.649 | 2.200 | 0.028 | 2.313 | 4.014 |
|  |  |  |  |  |  |  |
|  |  |  |  |  |  |  |
| **Outcome** | pCIS BATA | **Timepoint** | 2 Weeks |  |  |  |
|  | dy/dx | Delta-method | z | P>\|z\| | 95% conf. interval |  |
|  |  | std. err. |  |  |  |  |
| 0. Preparation | (base outcome) | | | | | |
| 1. Preperation |  | | | | | |
| Implant Surface |  |  |  |  |  |  |
| SLA | 39.847 | 8.463 | 4.710 | 0.000 | 23.259 | 56.434 |
| modSLA | 17.120 | 8.463 | 2.020 | 0.043 | 0.532 | 33.708 |
|  |  |  |  |  |  |  |
|  |  |  |  |  |  |  |
| **Outcome** | pCIS BATA | **Timepoint** | 8 Weeks |  |  |  |
|  | dy/dx | Delta-method | z | P>\|z\| | 95% conf. interval |  |
|  |  | std. err. |  |  |  |  |
| 0. Preparation | (base outcome) | | | | | |
| 1. Preperation |  | | | | | |
| Implant Surface |  |  |  |  |  |  |
| SLA | 11.735 | 6.750 | 1.740 | 0.082 | -1.496 | 24.965 |
| modSLA | 9.444 | 6.860 | 0.060 | 0.948 | -13.002 | 13.890 |

**Supplementary Table 3:** Linear Mixed Effect Model

| **Outcome** | fBIC (µm) | **Timepoint** | 2 weeks |  |  |  |
| --- | --- | --- | --- | --- | --- | --- |
|  |  |  |  |  |  |  |
|  | Coefficient | Std. err. | z | P>\|z\| | [95% conf. interval] | |
| Preparation OP | -534.68 | 155.66 | -3.43 | 0.001 | -839.77 | -229.58 |
| JawSide Right | 67.15 | 103.02 | 0.65 | 0.515 | -134.77 | 269.07 |
| Toothposition Middle Anterior | -158.76 | 125.53 | -1.26 | 0.206 | -404.80 | 87.27 |
| ImplantSurface modSLA | -91.41 | 103.02 | -0.89 | 0.375 | -293.34 | 110.51 |
| JawSide#Preparation Right#OP | -127.74 | 144.68 | -0.88 | 0.377 | -411.31 | 155.84 |
| ToothPosition#Preparation Middle Anterior#OP | 459.60 | 205.18 | 2.24 | 0.025 | 57.46 | 861.74 |
| Implant Surface#Preparation modSLA#OP | 420.25 | 144.68 | 2.9 | 0.004 | 136.68 | 703.83 |
| _cons | 650.49 | 111.22 | 5.85 | 0.000 | 432.51 | 868.47 |
|  |  |  |  |  |  |  |
|  |  |  |  |  |  |  |
| **Outcome** | fBIC (µm) | **Timepoint** | 8 weeks |  |  |  |
|  | Coefficient | Std. err. | z | P>\|z\| | [95% conf. interval] | |
| Preparation OP | -524.93 | 134.05 | -3.92 | 0.000 | -787.66 | -262.20 |
| JawSide Right | -183.86 | 87.42 | -2.1 | 0.035 | -355.19 | -12.53 |
| Toothposition Middle Anterior | -28.08 | 103.03 | -0.27 | 0.785 | -230.02 | 173.87 |
| ImplantSurface modSLA | -318.32 | 87.42 | -3.64 | 0.000 | -489.65 | -146.98 |
| JawSide#Preparation Right#OP | 134.22 | 123.27 | 1.09 | 0.276 | -107.39 | 375.83 |
| ToothPosition#Preparation Middle Anterior#OP | 14.71 | 163.64 | 0.09 | 0.928 | -306.02 | 335.43 |
| Implant Surface#Preparation modSLA#OP | 191.80 | 123.27 | 1.56 | 0.120 | -49.81 | 433.41 |
| _cons | 791.24 | 98.79 | 8.01 | 0.000 | 597.62 | 984.87 |
|  |  |  |  |  |  |  |
|  |  |  |  |  |  |  |
| **Outcome** | PCIS (µm) | **Timepoint** | 2 weeks |  |  |  |
|  | Coefficient | Std. err. | z | P>\|z\| | [95% conf. interval] | |
| Preparation OP | 201.43 | 72.53 | 2.78 | 0.005 | 59.28 | 343.58 |
| JawSide Right | -7.19 | 53.92 | -0.13 | 0.894 | -112.87 | 98.48 |
| Toothposition Middle Anterior | 16.26 | 53.92 | 0.3 | 0.763 | -89.41 | 121.94 |
| ImplantSurface modSLA | 44.22 | 53.92 | 0.82 | 0.412 | -61.45 | 149.90 |
| JawSide#Preparation Right#OP | -40.86 | 76.25 | -0.54 | 0.592 | -190.31 | 108.58 |
| ToothPosition#Preparation Middle Anterior#OP | -29.94 | 76.25 | -0.39 | 0.695 | -179.38 | 119.51 |
| Implant Surface#Preparation modSLA#OP | -95.16 | 76.25 | -1.25 | 0.212 | -244.61 | 54.29 |
| _cons | 102.23 | 51.28 | 1.99 | 0.046 | 1.71 | 202.75 |
|  |  |  |  |  |  |  |
| **Outcome** | PCIS (µm) | **Timepoint** | 8 weeks |  |  |  |
|  | Coefficient | Std. err. | z | P>\|z\| | [95% conf. interval] | |
| Preparation OP | -311.24 | 106.83 | -2.91 | 0.004 | -520.63 | -101.85 |
| JawSide Right | -194.49 | 70.00 | -2.78 | 0.005 | -331.69 | -57.30 |
| Toothposition Middle Anterior | -86.56 | 81.83 | -1.06 | 0.290 | -246.95 | 73.83 |
| ImplantSurface modSLA | -224.79 | 70.00 | -3.21 | 0.001 | -361.98 | -87.60 |
| JawSide#Preparation Right#OP | 200.47 | 98.73 | 2.03 | 0.042 | 6.96 | 393.97 |
| ToothPosition#Preparation Middle Anterior#OP | 65.69 | 129.33 | 0.51 | 0.611 | -187.78 | 319.16 |
| Implant Surface#Preparation modSLA#OP | 59.93 | 98.73 | 0.61 | 0.544 | -133.58 | 253.43 |
| _cons | 601.43 | 78.67 | 7.64 | 0.000 | 447.23 | 755.63 |
|  |  |  |  |  |  |  |
|  |  |  |  |  |  |  |
| **Outcome** | TotalBIC (%) | **Timepoint** | 2 weeks |  |  |  |
|  | Coefficient | Std. err. | z | P>\|z\| | [95% conf. interval] | |
| Preparation OP | -4.01 | 3.91 | -1.02 | 0.305 | -11.69 | 3.66 |
| JawSide Right | 3.76 | 2.91 | 1.29 | 0.196 | -1.94 | 9.46 |
| Toothposition Middle Anterior | 0.18 | 2.91 | 0.06 | 0.952 | -5.53 | 5.88 |
| ImplantSurface modSLA | -14.05 | 2.91 | -4.83 | 0.000 | -19.76 | -8.35 |
| JawSide#Preparation Right#OP | -0.61 | 4.12 | -0.15 | 0.881 | -8.68 | 7.45 |
| ToothPosition#Preparation Middle Anterior#OP | -12.28 | 4.12 | -2.98 | 0.003 | -20.34 | -4.21 |
| Implant Surface#Preparation modSLA#OP | -3.08 | 4.12 | -0.75 | 0.454 | -11.15 | 4.98 |
| _cons | 80.51 | 2.77 | 29.08 | 0.000 | 75.08 | 85.93 |
|  |  |  |  |  |  |  |
|  |  |  |  |  |  |  |
| **Outcome** | TotalBIC (%) | **Timepoint** | 8 weeks |  |  |  |
|  | Coefficient | Std. err. | z | P>\|z\| | [95% conf. interval] | |
| Preparation OP | -4.58 | 5.72 | -0.8 | 0.423 | -15.79 | 6.62 |
| JawSide Right | -1.30 | 3.78 | -0.34 | 0.731 | -8.70 | 6.10 |
| Toothposition Middle Anterior | -2.37 | 4.35 | -0.54 | 0.586 | -10.90 | 6.16 |
| ImplantSurface modSLA | -8.03 | 3.78 | -2.13 | 0.033 | -15.44 | -0.63 |
| JawSide#Preparation Right#OP | 3.30 | 5.33 | 0.62 | 0.536 | -7.15 | 13.74 |
| ToothPosition#Preparation Middle Anterior#OP | -2.23 | 6.82 | -0.33 | 0.744 | -15.59 | 11.13 |
| Implant Surface#Preparation modSLA#OP | 1.71 | 5.33 | 0.32 | 0.749 | -8.74 | 12.15 |
| _cons | 88.48 | 4.21 | 21.04 | 0.000 | 80.24 | 96.73 |
|  |  |  |  |  |  |  |
|  |  |  |  |  |  |  |
| **Outcome** | CoronalBIC (%) | **Timepoint** | 2 weeks |  |  |  |
|  | Coefficient | Std. err. | z | P>\|z\| | [95% conf. interval] | |
| Preparation OP | 41.29 | 10.22 | 4.04 | 0.000 | 21.25 | 61.33 |
| JawSide Right | 11.48 | 6.94 | 1.66 | 0.098 | -2.12 | 25.08 |
| Toothposition Middle Anterior | 6.86 | 8.12 | 0.84 | 0.399 | -9.06 | 22.78 |
| ImplantSurface modSLA | -5.41 | 6.94 | -0.78 | 0.436 | -19.01 | 8.19 |
| JawSide#Preparation Right#OP | -13.66 | 9.76 | -1.4 | 0.162 | -32.79 | 5.47 |
| ToothPosition#Preparation Middle Anterior#OP | -19.81 | 12.99 | -1.52 | 0.127 | -45.26 | 5.65 |
| Implant Surface#Preparation modSLA#OP | -13.63 | 9.76 | -1.4 | 0.162 | -32.76 | 5.49 |
| _cons | 23.80 | 7.28 | 3.27 | 0.001 | 9.54 | 38.07 |
|  |  |  |  |  |  |  |
|  |  |  |  |  |  |  |
| **Outcome** | CoronalBIC (%) | **Timepoint** | 8 weeks |  |  |  |
|  | Coefficient | Std. err. | z | P>\|z\| | [95% conf. interval] | |
| Preparation OP | 42.86 | 15.21 | 2.82 | 0.005 | 13.04 | 72.68 |
| JawSide Right | 4.11 | 10.76 | 0.38 | 0.702 | -16.98 | 25.20 |
| Toothposition Middle Anterior | -5.40 | 10.93 | -0.49 | 0.621 | -26.82 | 16.03 |
| ImplantSurface modSLA | 14.87 | 10.76 | 1.38 | 0.167 | -6.22 | 35.96 |
| JawSide#Preparation Right#OP | 0.32 | 15.21 | 0.02 | 0.983 | -29.50 | 30.14 |
| ToothPosition#Preparation Middle Anterior#OP | 7.53 | 15.46 | 0.49 | 0.626 | -22.77 | 37.82 |
| Implant Surface#Preparation modSLA#OP | -20.34 | 15.21 | -1.34 | 0.181 | -50.16 | 9.48 |
| _cons | 35.83 | 11.10 | 3.23 | 0.001 | 14.08 | 57.59 |
|  |  |  |  |  |  |  |
|  |  |  |  |  |  |  |
| **Outcome** | ApicalBIC (%) | **Timepoint** | 2 weeks |  |  |  |
|  | Coefficient | Std. err. | z | P>\|z\| | [95% conf. interval] | |
| Preparation OP | -1.18 | 6.57 | -0.18 | 0.858 | -14.05 | 11.69 |
| JawSide Right | -0.67 | 4.88 | -0.14 | 0.890 | -10.24 | 8.89 |
| Toothposition Middle Anterior | 2.26 | 4.88 | 0.46 | 0.643 | -7.31 | 11.83 |
| ImplantSurface modSLA | -3.40 | 4.88 | -0.7 | 0.487 | -12.96 | 6.17 |
| JawSide#Preparation Right#OP | 4.46 | 6.90 | 0.65 | 0.518 | -9.07 | 17.99 |
| ToothPosition#Preparation Middle Anterior#OP | -15.10 | 6.90 | -2.19 | 0.029 | -28.63 | -1.57 |
| Implant Surface#Preparation modSLA#OP | -14.79 | 6.90 | -2.14 | 0.032 | -28.33 | -1.26 |
| _cons | 77.39 | 4.64 | 16.67 | 0.000 | 68.29 | 86.49 |
|  |  |  |  |  |  |  |
|  |  |  |  |  |  |  |
| **Outcome** | ApicalBIC (%) | **Timepoint** | 8 weeks |  |  |  |
|  | Coefficient | Std. err. | z | P>\|z\| | [95% conf. interval] | |
| Preparation OP | 2.13 | 5.85 | 0.36 | 0.716 | -9.35 | 13.60 |
| JawSide Right | 4.11 | 3.49 | 1.18 | 0.238 | -2.72 | 10.95 |
| Toothposition Middle Anterior | -1.67 | 4.76 | -0.35 | 0.725 | -11.00 | 7.65 |
| ImplantSurface modSLA | -1.04 | 3.49 | -0.3 | 0.767 | -7.87 | 5.80 |
| JawSide#Preparation Right#OP | -6.50 | 4.90 | -1.33 | 0.184 | -16.11 | 3.10 |
| ToothPosition#Preparation Middle Anterior#OP | 2.82 | 8.11 | 0.35 | 0.728 | -13.08 | 18.72 |
| Implant Surface#Preparation modSLA#OP | -9.70 | 4.90 | -1.98 | 0.048 | -19.30 | -0.10 |
| _cons | 78.18 | 4.40 | 17.78 | 0.000 | 69.56 | 86.80 |
|  |  |  |  |  |  |  |
|  |  |  |  |  |  |  |
| **Outcome** | BATA (%) | **Timepoint** | 2 weeks |  |  |  |
|  | Coefficient | Std. err. | z | P>\|z\| | [95% conf. interval] | |
| Preparation OP | 35.30 | 8.78 | 4.02 | 0.000 | 18.09 | 52.51 |
| JawSide Right | -3.40 | 5.54 | -0.61 | 0.539 | -14.27 | 7.47 |
| Toothposition Middle Anterior | 21.92 | 7.26 | 3.02 | 0.003 | 7.69 | 36.15 |
| ImplantSurface modSLA | -20.99 | 5.54 | -3.78 | 0.000 | -31.85 | -10.12 |
| JawSide#Preparation Right#OP | 8.86 | 7.76 | 1.14 | 0.253 | -6.35 | 24.08 |
| ToothPosition#Preparation Middle Anterior#OP | -38.50 | 12.28 | -3.14 | 0.002 | -62.56 | -14.44 |
| Implant Surface#Preparation modSLA#OP | 1.50 | 7.76 | 0.19 | 0.847 | -13.71 | 16.71 |
| _cons | 42.60 | 6.33 | 6.73 | 0.000 | 30.19 | 55.01 |
|  |  |  |  |  |  |  |
|  |  |  |  |  |  |  |
| **Outcome** | BATA (%) | **Timepoint** | 8 weeks |  |  |  |
|  | Coefficient | Std. err. | z | P>\|z\| | [95% conf. interval] | |
| Preparation OP | 28.54 | 13.54 | 2.11 | 0.035 | 2.01 | 55.08 |
| JawSide Right | 10.61 | 9.57 | 1.11 | 0.268 | -8.16 | 29.37 |
| Toothposition Middle Anterior | 6.59 | 9.73 | 0.68 | 0.498 | -12.47 | 25.65 |
| ImplantSurface modSLA | -6.28 | 9.57 | -0.66 | 0.512 | -25.04 | 12.48 |
| JawSide#Preparation Right#OP | 5.36 | 13.54 | 0.4 | 0.692 | -21.18 | 31.89 |
| ToothPosition#Preparation Middle Anterior#OP | -12.72 | 13.75 | -0.92 | 0.355 | -39.67 | 14.24 |
| Implant Surface#Preparation modSLA#OP | -3.64 | 13.54 | -0.27 | 0.788 | -30.17 | 22.89 |
| _cons | 25.71 | 9.88 | 2.6 | 0.009 | 6.35 | 45.06 |
|  |  |  |  |  |  |  |
|  |  |  |  |  |  |  |
| **Outcome** | PCIS BATA (%) | **Timepoint** | 2 weeks |  |  |  |
|  | Coefficient | Std. err. | z | P>\|z\| | [95% conf. interval] | |
| Preparation OP | 51.70 | 11.52 | 4.49 | 0.000 | 29.12 | 74.28 |
| JawSide Right | 2.92 | 8.57 | 0.34 | 0.733 | -13.87 | 19.71 |
| Toothposition Middle Anterior | 3.73 | 8.57 | 0.44 | 0.663 | -13.06 | 20.52 |
| ImplantSurface modSLA | -1.12 | 8.57 | -0.13 | 0.896 | -17.90 | 15.67 |
| JawSide#Preparation Right#OP | -11.89 | 12.11 | -0.98 | 0.326 | -35.63 | 11.85 |
| ToothPosition#Preparation Middle Anterior#OP | -11.82 | 12.11 | -0.98 | 0.329 | -35.56 | 11.93 |
| Implant Surface#Preparation modSLA#OP | -22.73 | 12.11 | -1.88 | 0.061 | -46.47 | 1.02 |
| _cons | 6.93 | 8.15 | 0.85 | 0.395 | -9.04 | 22.90 |
|  |  |  |  |  |  |  |
|  |  |  |  |  |  |  |
| **Outcome** | PCIS BATA (%) | **Timepoint** | 8 weeks |  |  |  |
|  | Coefficient | Std. err. | z | P>\|z\| | [95% conf. interval] | |
| Preparation OP | 17.78 | 9.62 | 1.85 | 0.065 | -1.08 | 36.64 |
| JawSide Right | 8.20 | 6.81 | 1.2 | 0.228 | -5.14 | 21.54 |
| Toothposition Middle Anterior | 1.43 | 6.91 | 0.21 | 0.836 | -12.12 | 14.99 |
| ImplantSurface modSLA | -5.27 | 6.81 | -0.77 | 0.439 | -18.60 | 8.07 |
| JawSide#Preparation Right#OP | -4.30 | 9.62 | -0.45 | 0.655 | -23.16 | 14.56 |
| ToothPosition#Preparation Middle Anterior#OP | -7.79 | 9.78 | -0.8 | 0.426 | -26.96 | 11.38 |
| Implant Surface#Preparation modSLA#OP | -11.29 | 9.62 | -1.17 | 0.241 | -30.15 | 7.57 |
| _cons | 10.42 | 7.02 | 1.48 | 0.138 | -3.34 | 24.18 |
